# Peak V̇O_2_ and Ventilatory threshold of male college basketball players: A sport‐specific protocol for prediction, evaluation and application

**DOI:** 10.14814/phy2.70499

**Published:** 2025-10-18

**Authors:** Brian D. Duscha, William C. Bennett, Jose Fonseca, Nicholas Potter, Kelsey N. Belski, Megan A. Reaves, Brian J. Coyne, Annunziato Amendola, Aaron L. Baggish, William E. Kraus

**Affiliations:** ^1^ Division of Cardiology Duke School of Medicine and Duke University Durham North Carolina USA; ^2^ Duke Molecular Physiology Institute Duke School of Medicine and Duke University Durham North Carolina USA; ^3^ Duke Sports Sciences Institute Duke School of Medicine and Duke University Durham North Carolina USA; ^4^ Athletic Medicine Duke School of Medicine and Duke University Durham North Carolina USA; ^5^ Division of Cardiology Massachusetts General Hospital Boston Massachusetts USA; ^6^ Cardiovascular Performance Program Massachusetts General Hospital Boston Massachusetts USA; ^7^ Swiss Olympic Medical Center Lausanne University Hospital (CHUV) Lausanne Switzerland; ^8^ Institute for Sport Science University of Lausanne (ISSUL) Lausanne Switzerland

**Keywords:** aerobic fitness, cardiopulmonary exercise testing, Duke basketball treadmill protocol, fitness equation

## Abstract

Although basketball is not an endurance sport, a high level of peak oxygen consumption (peak V̇O_2_) and ventilatory threshold (VT) are beneficial. Currently, no validated cardiopulmonary exercise test (CPET) protocols exist for basketball. This study developed and validated a basketball‐specific CPET protocol and describes the fitness measures. Sixty treadmill CPET's were performed on NCAA Division 1 basketball players. The first 30 CPET's created a prediction equation for peak V̇O_2_, and the second 30 tested the validity of the equation. The equation, *y* = 2.70x + 24.84; *r* ^2^ = 0.995, predicted peak V̇O_2_. Mean relative peak V̇O_2_ was 54.1 ± 4.6 mL min^−1^ kg^−1^. Mean relative V̇O_2_ at VT was 37.8 ± 5.8 mL min^−1^ kg^−1^, and heart rate (HR) at VT was 155.1 ± 14.2 bpm. These corresponded to 70.1 ± 10.7% of peak V̇O_2_, and 81.4 ± 6.3% of peak HR values. This equation can accurately determine oxygen consumption when direct metabolic testing is not available. The measures of peak V̇O_2_, VT, and HRs can be used to assess player fitness, design training protocols, and potentially help explain in‐game performance. Additional research is needed to fully establish the relationship between CPET values and basketball performance.

## 1 INTRODUCTION

Peak oxygen consumption (peak V̇O_2_) and ventilatory threshold (VT) are two important determinations of cardiorespiratory fitness (CRF) (Beaver et al., 1985; Hill et al., 1924). There are descriptive studies reporting fitness levels in sedentary individuals (Kaminsky et al., 2022; Loe et al., 2013; Wasserman et al., 2005), non‐endurance sports (Feairheller et al., 2016; Gillet et al., 2016; Shields et al., 1984; Wilmore & Haskell, 1972; Yang, 2014), mixed sports involving aerobic and anaerobic activity (Bergeron et al., 1991; Crisp et al., 2013; Gilenstam et al., 2011; Green et al., 2006; Lowery et al., 2018; Raven et al., 1976; Santos‐Silva et al., 2017; Smekal et al., 2001) and, to a larger degree, endurance sports (Billat et al., 2002; Billat et al., 2003; Bodner et al., 2002; Brandon, 1995; Costill & Fox, 1969; Driller et al., 2013; Helgerud, 1994; Hoogeveen et al., 1999; Kimura et al., 1990; Kumagai et al., 1982; Mickelson & Hagerman, 1982; Reis et al., 2012; Roels et al., 2005; Yoshiga & Higuchi, 2003). Basketball is a widely played international sport, having elite levels inclusive of college, professional, and Olympic tiers. Despite the value of a cardiopulmonary exercise test (CPET), there are very few large studies using a treadmill and a metabolic cart to directly measure peak V̇O_2_ in elite basketball players— and fewer studying VT in this population. Given the importance of a pre‐season physical examination and the value of identifying an athlete's fitness level, it may be highly beneficial for a basketball team to perform a CPET during the pre‐season.

In addition to evaluating pre‐season peak V̇O_2_, coaches, trainers, and medical staff might find a CPET valuable for other reasons. A CPET allows comparison of fitness from year to year; serves as a reference value in the event of an unexpected illness or injury; serves as an objective reference for the performances observed at practice or in games (unexplained fatigue vs. effort); provides important information for exercise prescription (e.g., individual peak HRs and HR at VT); and determines the effects of a training regimen. Knowledge of individual player VT and recovery times may potentially forecast playing minutes and strategic substitutions within a game. Although an increasing number of basketball teams are gaining access to cardiopulmonary exercise testing with gas analysis, there remains a significant number of teams that do no. Therefore, a treadmill protocol, with an associated metabolic equation to accurately predict oxygen consumption, would be a highly useful tool. To our knowledge, there is no validated basketball player‐specific treadmill protocol established to accurately assess oxygen consumption. The purpose of this study was to develop and validate a protocol‐specific treadmill test for elite basketball players, describe the results, and compare results to previous reports.

## 2 METHODS

### 2.1 Subjects

Male basketball players from an NCAA Division I team were tested during the pre‐season for six consecutive years (2017–2022). A total of 60 tests were included from 40 different players. Because some players were on the roster for up to 4 years, 14 players had multiple tests during their tenure. Each year, all players on the roster were tested, with the exclusion of players with current injuries or due to the coach's discretion. Subjects had no cardiopulmonary dysfunction as indicated by history and physical examination, and none exhibited symptoms of ischemic heart disease or clinically concerning heart rhythm by exercise or ECG tracing. No subject was taking a prescription medication that would affect a cardiovascular response, nor did any subject have a comorbid illness inclusive of diabetes, COPD, hypertension, obesity, hematologic disorder, or infection. No special instructions were provided in the weeks leading up to testing, and the players were following their usual off‐season physical activity regimens. During each of the six consecutive years, all tests were performed in July or August, within a week of the athlete's arrival on campus. Four athletes were excluded because their age was <18 years old at the time of testing; two tests were excluded from the analysis because the CPET was terminated prior to maximum (muscle tightness, back pain); five tests had either a mouthpiece leak or nose clip issue yielding inaccurate metabolic data for a portion of the CPET. All subjects were informed of testing protocols and the potential risks and benefits of participation. Each subject provided written informed consent before enrollment in the study. The Institutional Review Board of Duke University approved the research protocol.

### 2.2 Cardiopulmonary exercise testing (CPET)

Subjects underwent a maximal CPET with a 12‐lead ECG and expired gas analysis on a calibrated treadmill. Expired gases were analyzed continuously using a TrueMax 2400 Parvo Medics unit (Sandy, UT, USA). The Duke Basketball Treadmill Protocol (DBTP) (Table 1) consists of an initial two stages of 2 min, followed by 1‐min stages, with a goal of increasing oxygen consumption by 2–4 mL min^−1^ kg^−1^ per stage. The same metabolic cart and treadmill were used across all subjects. Subjects were asked to run to volitional fatigue; the rate of perceived exertion (RPE) was documented every 2–3 min and at peak exercise. Expired gases were analyzed every 15 s. The last 30 s were averaged to determine peak V̇O_2_. In addition, peak V̇O_2_ was estimated using the American College of Sports Medicine metabolic equation for running (ACSM 2013) (American College of Sports Medicine, 2013) and the Wasserman equation (Wasserman et al., 2005). Tests 1–30 were used to develop a regression equation to predict oxygen consumption at any time point during the CPET. Tests 31–60 were used to compare directly measured peak V̇O_2_ to the prediction equation.

**TABLE 1:** Duke basketball treadmill protocol (DBTP)^a^.

Stage	Min	Speed (mph)	Elevation (%)	ACSM calculated^a^ V̇O_2_ (mL min^−1^ kg^−1^)	Measured V̇O_2_ (mL min^−1^ kg^−1^)	DBTP calculated V̇O_2_ (mL min^−1^ kg^−1^)^b^
1	1‐2	6.0	0	35.6	31.2 ± 3.4	31.2
2	3–4	6.0	2.0	38.5	34.4 ± 2.2	33.9
3	5	6.0	4.0	38.5	37.0 ± 2.5	36.6
4	6	6.5	4.0	41.4	39.4 ± 2.5	39.2
5	7	7.0	4.0	44.7	42.1 ± 2.6	42.0
6	8	7.0	6.0	47.7	45.2 ± 2.5	44.7
7	9	7.5	6.0	51.1	47.8 ± 2.7	47.3
8	10	7.5	8.0	54.5	50.6 ± 2.9	50.0
9	11	8.0	8.0	58.1	53.1 ± 3.2	52.7
10	12	8.0	10.0	61.8	56.1 ± 3.0	55.4
11	13	8.5	10.0	65.6	57.8 ± 1.1	58.1
12	14	8.5	11.0	69.5	61.9 ± 1.8	60.8

^a^ Calculated from ACSM metabolic equations; V̇O_2_ (0.2 × speed) × (0.9× grade) + 3.5.

^b^ From Duke Protocol regression equation; y = 2.70x + 24.84.

### 2.3 Determination of Ventilatory threshold (VT)

Ventilatory threshold was determined using the V‐slope method (Pollock et al., 1976). Fifteen‐second averages of V̇O_2_ and V̇CO_2_ were obtained from the metabolic cart. Two separate experienced readers were given, in random order, unidentified plots of V̇O_2_ versus V̇CO_2_ production for each CPET and asked to mark the point of VT. For a value to be considered valid, both readers had to agree within a variance of 150 mL min^−1^ of oxygen. If the two readers agreed, the values were averaged. If the two readers were not in agreement within 150 mL min^−1^, a third experienced reader blindly read the plot. The third reader had to agree with one of the two original readers within the parameter of 150 mL min^−1^. If no two of three readers failed to agree within 150 mL min^−1^, the V̇O_2_ at VT was considered indeterminate.

### 2.4 Statistical analysis

Means and standard deviations were used to describe player demographics. Differences in demographic and performance characteristics between academic years in school were determined using a one‐way ANOVA and a Bonferroni post‐hoc test. Linear regression between each minute of the progressive CPET test and measured oxygen consumption was used to derive a metabolic prediction equation. All tests were two‐tailed. Tabular data are presented as means ± SD, and in figures as ± SE. *p* values of ≤0.05 were considered significant for all tests. Data analysis was performed using the Statistical Package for the Social Sciences (SPSS) version 28 (SPSS, Inc., Chicago, Illinois, USA).

## 3 RESULTS

### 3.1 Demographics

Demographics are shown in Table 2. For statistical reasons, two fifth‐year seniors (graduate transfers) were included with six fourth‐year seniors, making eight senior subjects. There were no differences in height, weight, BMI, resting HR, or blood pressure across the years in school; all values represented expected values for NCAA Division 1 basketball players.

**TABLE 2:** Subject demographics (mean ± SD).

School year	*N*	Age (years)	Race (Cauc./A.A.)	Height (cm)	Weight (kg)	BMI (kg m^−1^)	Heart rate (bpm)	Blood pressure (mmHg)
Freshman	24	18.1 ± 0.3	6/18	199.8 ± 9.2	94.7 ± 13.2	23.6 ± 2.1	68.0 ± 12.8	117.0 ± 09.3 73.6 ± 7.8
Sophomore	14	19.2 ± 0.6^†^	7/7	194.3 ± 10.5	90.7 ± 12.8	23.9 ± 1.9	69.8 ± 12.3	119.1 ± 11.9 71.3 ± 8.4
Junior	14	20.1 ± 0.5^†^	7/7	197.8 ± 8.4	94.5 ± 13.0	24.0 ± 1.7	64.1 ± 11.6	120.1 ± 10.1 71.7 ± 11.7
Senior^a^	8	22.0 ± 0.9^†^	3/5	198.8 ± 8.3	93.7 ± 10.4	23.6 ± 1.1	65.6 ± 11.6	117.5 ± 8.3 73.5 ± 6.3
Total	60	19.3 ± 1.4	23/42	197.9 ± 9.3	93.6 ± 12.5	23.8 ± 1.8	67.2 ± 12.1	118.3 ± 9.9 72.6 ± 8.7

*Note*: Underlined values illustrate systoic blood pressure/diastolic blood pressure.

Abbreviations: A.A./Cauc. = African American/Caucasian, BMI = Body Mass Index, BP = blood pressure.

^a^ Six four‐year seniors and two fifth‐year graduate transfer seniors.

^†^ *p* < 0.001 vs all other groups.

### 3.2 Comparison of the Duke basketball treadmill protocol (DBTP) equation to directly measured oxygen consumption

**FIGURE 1:**
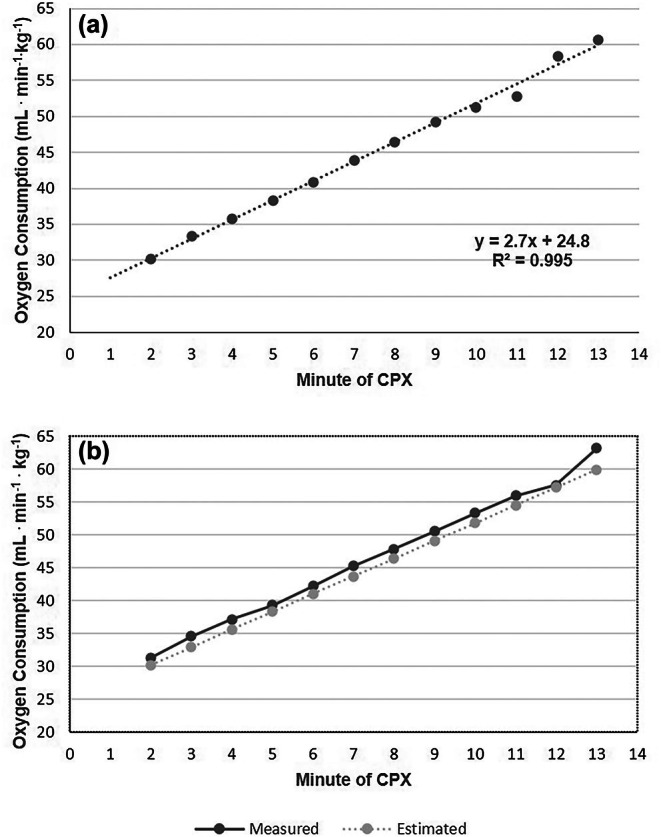
Derived regression equation from first 30 CPET Tests (Panel A) and overlay of measured oxygen consumption versus derived oxygen consumption using CPET Tests from 30 different CPET tests (Panel B).

The mean increase per stage of the protocol was 2.8 ± 0.6 mL min^−1^ kg^−1^; this was within our goal of 2–4 mL min^−1^ kg^−1^. Figure 1a shows the V̇O_2_ regression line derived from the mean of each minute of the CPET test for tests 1–30. From these data, the DBTP prediction equation for oxygen consumption was established as *y* = 2.70*x* + 24.84; where × represents the exact time at any point in the CPET (e.g., 8:30 equates to 8.5). The *r* ^2^ for this was 0.995. Figure 1b shows the validation of this by overlaying the oxygen consumption from the equation (estimated) to the direct measurement of oxygen consumption on data from CPET tests 31–60. The mean difference of oxygen consumption between methods for each minute (measured vs. predicted) was 1.5 mL min^−1^ kg^−1^ (3.3%).

### 3.3 Comparison of the Duke basketball treadmill protocol (DBTP) equation, the American College of Sports Medicine (ACSM) equation, the Wasserman equation, and directly measured peak oxygen consumption

**FIGURE 2:**
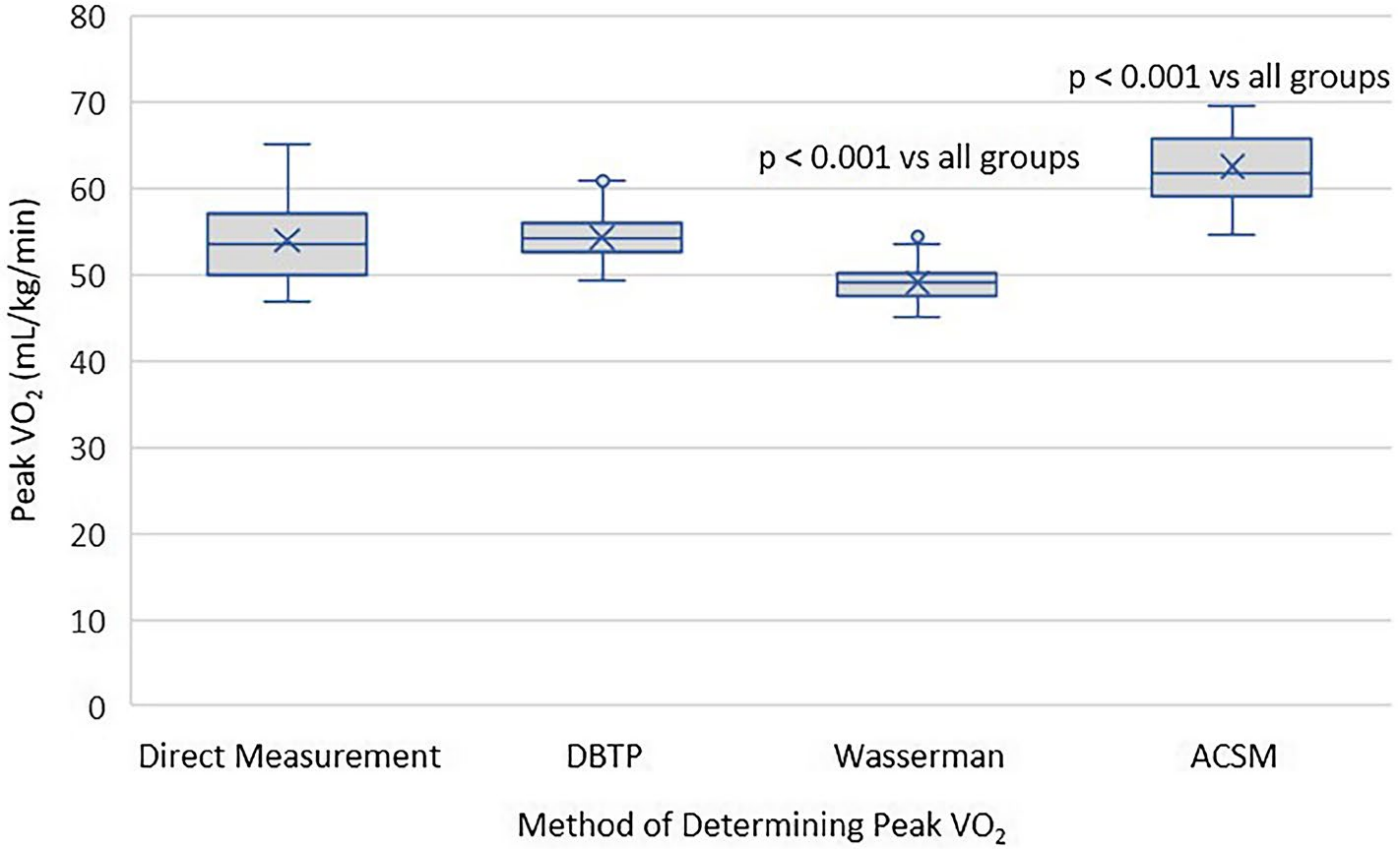
Box plot comparison of methods to determine peak V̇O_2_ with outliers. mean values are represented by (X), median by (^___^), outliers by (°), and bars represent minimal and maximal values.

The mean ± SD mL kg min for the four methods was as follows: DBTM 54.3 ± 2.8, ACSM 62.5 ± 3.9, Wasserman 49.1 ± 2.1, and direct measurement 54.1 ± 4.6. To better represent the data distribution, Figure 2 compares the peak V̇O_2_ among the four methods by a box plot. The directly measured peak V̇O_2_ and predictive equation from the DBTP were not significantly different. Both the Wasserman equation and the ACSM equation was different compared to all other methods.

### 3.4 Peak V̇O_2_ and Ventilatory threshold

Subject effort and true maximal tests were ensured by peak respiratory exchange ratio (RER) and rate of perceived exertion (RPE). Ninety percent of all tests (*n* = 54) reached a peak RER of ≥1.05, with the mean for the entire 60 tests of 1.13 ± 0.06. Five subjects had a peak RER of 1.03 or 1.04, and one subject had an RER = 0.98, but a RPE of 19. Mean peak RPE was 18.4 ± 1.0.

Table 3 shows the peak V̇O_2_, peak HR, and heart rate recovery (HRR) values for all players. Mean relative and absolute peak V̇O_2_ was 54.1 ± 4.6 mL min^−1^ kg^−1^ and 5.0 ± 0.5 L min^−1^ for all players combined. The percent predicted peak V̇O_2_ was greater for seniors compared to freshmen (118.9 ± 7.0 vs. 106.5 ± 5.6%; *p* < 0.05) as was the percent predicted peak HR (98.9 ± 6.1 vs. 92.6 ± 4.2; *p* < 0.05). There were no differences detected across school years for peak HR or HRR values. Table 4 shows VT values. Mean relative V̇O_2_ at VT was 37.8 ± 5.8 mL min^−1^ kg^−1^ and HR at VT was 155.1 ± 14.2 bpm among all players combined. These corresponded to 70.1 ± 10.7% of peak V̇O_2_ and 81.4 ± 6.3% of peak values.

**TABLE 3:** Peak V̇O_2_, heart rate (HR), and heart rate recovery (HRR) (mean ± SD).

Year	Peak V̇O_2_ (mL min^−1^ kg^−1^)	Peak V̇O_2_ (L min^−1^)	Predicted^a^ peak V̇O_2_ (%)	Peak HR (bpm)	Predicted peak HR (%)	1 min HRR (bpm)	2 min HRR (bpm)
Freshman	52.4 ± 4.0	4.9 ± 0.5	106.5 ± 5.6^b^	187.5 ± 8.5	92.6 ± 4.2^b^	32.6 ± 11.8	63.2 ± 13.9
Sophomore	55.2 ± 5.2	5.0 ± 0.5	112.8 ± 8.5	191.3 ± 7.5	95.3 ± 3.9	26.6 ± 12.2	59.9 ± 17.9
Junior	54.2 ± 4.5	5.1 ± 0.6	112.7 ± 8.2	192.5 ± 10.3	96.3 ± 5.3	36.8 ± 15.6	64.9 ± 12.2
Senior	57.0 ± 4.0	5.4 ± 0.6	118.9 ± 7.00	195.9 ± 12.3	98.9 ± 6.1	29.4 ± 12.4	62.7 ± 10.4
Total	54.1 ± 4.6	5.0 ± 0.5	111.1 ± 8.2	190.7 ± 9.5	94.9 ± 5.1	31.8 ± 13.1	62.8 ± 14.0

^a^ Based on Wasserman equation (Wasserman et al., 2005).

^b^ *p* < 0.05 Freshman vs. Seniors.

**TABLE 4:** Oxygen consumption (V̇O_2_) and heart rate (HR) at ventilatory threshold (VT) (mean ± SD).

School year	V̇O_2_ at VT (mL min^−1^ kg^−1^)	VT (% peak V̇O_2_)	HR at VT (bpm)	VT (% peak HR)
Freshman	37.7 ± 5.8	71.6 ± 10.1	156.5 ± 15.8	83.4 ± 6.5
Sophomore	37.1 ± 5.7	67.9 ± 10.3	154.4 ± 13.1	80.7 ± 5.7
Junior	38.5 ± 6.9	71.2 ± 13.1	155.6 ± 14.1	80.1 ± 7.9
Senior	38.2 ± 4.9	67.2 ± 8.8	151.4 ± 12.6	77.3 ± 4.4
Total	37.8 ± 5.8	70.1 ± 10.7	155.1 ± 14.2	81.4 ± 6.3

## 4 DISCUSSION

To our knowledge, this is the largest (*N* = 60) study using a specific treadmill protocol and a metabolic cart for direct measurement of expired gases to evaluate elite‐level basketball players for both peak V̇O_2_ and VT. Numerous treadmill protocols have been developed that elicit a true peak V̇O_2_ and HR in middle‐aged healthy subjects (Green et al., 2023; Pollock et al., 1976). Although studies show commonly used protocols do not demonstrate a significant difference between measured peak V̇O_2_ and HR, differences do exist in time to exhaustion, maximal ventilation, and respiratory exchange ratio (Green et al., 2023; Pollock et al., 1976). More important to testing athletic populations, these differences widen when comparing sedentary and active subjects (Pollock et al., 1976). There is evidence a protocol should initiate volitional fatigue between 8:00 and 15:00 min (Buchfuhrer et al., 1983). If too aggressive of a protocol is chosen, greater localized metabolic acidosis may occur. This will cause early fatigue prior to maximal recruitment of the entire cardiorespiratory and muscular systems. In contrast, if a protocol is too passive for an athlete, a longer than desired time to fatigue may occur potentially lowering peak V̇O_2_. One potential reason for this is that longer times may contribute to increased core temperatures and peripheral vasodilation causing a reduced blood volume for peak cardiac output. The combination of an erroneously low peak V̇O_2_ and too fast of a rise in gas kinetics would greatly affect VT.

### 4.1 Appropriateness of Duke basketball treadmill protocol

Currently, there is no validated basketball‐specific treadmill protocol to determine peak V̇O_2_. It is important to choose a CPET exercise testing protocol and modality specific to the population being studied (e.g., cyclist on a cycle ergometer). Although basketball is a sport of intermittent running, a continuous treadmill protocol was chosen as running is the modality that best represents playing basketball, and intermittent running would be difficult to determine peak V̇O_2_ and VT. The protocol's mean time to volitional fatigue was 10:33 min, with minimal‐maximal times of 8:43–13:02 min. Therefore, this protocol met the criteria of an 8:00–15:00‐min window. Prior to testing, the goal was to increase the workload in stages that would initiate a gradual increase of 2–4 mL min^−1^ kg^−1^ of oxygen. The protocol was designed to allow a gradual HR increase and to plateau without a large increase in the final stage. The data show that both a gradual increase in oxygen consumption (2.80 mL min^−1^ kg^−1^) and HR (8–10 bpm) were achieved. The mean peak RER for all 60 tests was 1.13 ± 0.06. Mean peak RPE was 18.4 ± 1.0. Lastly, all players reported symptoms of generalizable volitional fatigue. Therefore, all tests met pre‐specified end points for true peak V̇O_2_.

### 4.2 Duke basketball treadmill protocol predictive equation and validation

An over‐lay of the best‐fit line for both the regression equation for estimating oxygen consumption (CPET tests 1–30) and direct measurement (CPET tests 31–60) is shown in Figure 1b. Our results indicate the predictive equation of *y* = 2.70*x* + 24.84; *r* ^2^ = 0.995 is highly accurate in estimating oxygen consumption at any time during the test. Statistically, this implies that the equation to predict oxygen consumption explains approximately 99% of cases within two standard deviations. The mean difference of oxygen consumption between methods for each minute (measured vs. predicted) was 1.5^−1^ mL min kg^−1^ (3.3%) up to 13 min (no player went beyond 13 min). The estimated peak V̇O_2_ was 53.4 ^−1^mL min kg^−1^ compared to 54.1 ^−1^mL min kg^−1^ for the direct measurement of peak V̇O_2_ (a difference of 1.3%). Only one study, Parpa et al., that we are aware of reported a regression equation for prediction of peak V̇O_2_. The authors did not directly compare the directly measured peak V̇O_2_ with the peak V̇O_2_ from the regression equation for accuracy. The coefficient of determination (*r* ^2^) was 0.68. The current study's *r* ^2^ for predicting peak V̇O_2_ was 0.99, suggesting the regression model may be superior.

The ACSM equation for running appears to overestimate oxygen consumption (Dugas et al., 2021; Kokkinos et al., 2017; Koutlianos et al., 2013; Ruiz & Sherman, 1999). In the current study, the ACSM calculation overestimated the V̇O_2_ at all workloads by an average of 4.2 ± 2.0 mL min^−1^ kg^−1^ (8.9 ± 3.4%) compared to the direct measurement of V̇O_2_. Figure 2 shows that the ACSM equation also overestimated peak V̇O_2_ by 8.4 mL min^−1^ kg^−1^, or 15.6%, versus direct measurement. This was much more than at any single sub‐maximal time point. This likely occurs because the assumption of the equation is that the subject completes the full workload. However, it takes 30–60 s to observe the true increase of V̇O_2_ after an increase in speed and grade. Therefore, if the subject terminates a stage after only 10–15 s into that stage, it is unlikely their V̇O_2_ will represent the full metabolic requirement of that stage–which is more reflective of the ACSM equation at peak. The Wasserman equation significantly underestimated the peak V̇O_2_ by 5 mL min^−1^ kg^−1^, or 9.2%. Therefore, both the ACSM and the Wasserman equation were inaccurate compared to both the directly measured and the DBTP, but in opposite directions. Because the values could be as much as 25% different at peak exercise, this becomes problematic when comparing studies of the same population when using two different predictive equations. A major advantage of the current predictive equation compared to others is the homogeneity of the population. In the current study, all players were men, between 18 and 25 years of age, and had similar fitness levels. Based on these data, teams evaluating young elite basketball players, not able to utilize a metabolic cart, should consider using this method.

### 4.3 Peak V̇O_2_ and VT comparison to previous studies

Because large databases describe healthy populations by means of decades starting at 20 years of age (e.g., 20–30 years old average), it is difficult to compare peak V̇O_2_ of our subjects to the normal population. The mean age of the current study was 19.3 ± 1.4 years with 35 of 60 (58.3%) tests representing players less than 20 years old. The measured peak V̇O_2_ of 54.1 ± 4.6^−1^ mL min kg^−1^ of NCAA Division 1 basketball players in this investigation is approximately 15%–20% above average compared to non‐competitive individuals between 20 and 30 years old in the United States, but similar to age‐matched individuals in Europe compared to available registries (Kaminsky et al., 2022; Loe et al., 2013). The oxygen consumption at VT was 37.8 ± 5.8^−1^ mL min kg^−1^ (70% of peak V̇O_2_), also greater than non‐athlete values of 40%–65% of peak V̇O_2_ (Wasserman et al., 2005).

We found several studies of elite basketball players using a treadmill with direct measurement of oxygen consumption via a metabolic cart for comparison of peak V̇O_2_. These studies (Apostolidis et al., 2004; Bolonchuk et al., 1991; Boone & Bourgois, 2013; Caterisano et al., 1997; Crisp et al., 2013; Lazic et al., 2019; McInnes et al., 1995; Metaxas et al., 2009; Narazaki et al., 2009; Parpa & Michaelides, 2022; Parr et al., 1978; Ponce‐González et al., 2015; Sallet et al., 2005) are depicted in Table 5. In these 13 studies of elite basketball players of the same sex and similar age, the range of peak V̇O_2_ is 46.0 ± 4.7–60.7 ± 8.6 mL kg min^−1^. Although there is a range of CRF within individual basketball players, it is unlikely means of cohorts would be this wide between studies of similar players (all men, young, basketball players). Potential reasons for this large range may be training status at the time of testing, maximal exertion levels, and different protocols. The heterogeneity of the protocols makes objective comparisons difficult, especially when time to exhaustion, time to VT, and rate of oxygen uptake are not reported. It is possible the amount of time spent at a low intensity or a more gradual grade elevation may influence peak V̇O_2_or VT. Table 5 includes workloads at the 5:00 and 10:00 mark of different studies along with peak V̇O_2_ and VT. Due to a lack of power and information across all studies, a statistical comparison on protocol influence could not be done. Studies measuring VT and peak V̇O_2_, using different protocols, on the same player are necessary to answer comparative protocol questions.

**TABLE 5:** Basketball studies directly measuring peak V̇O_2_ and ventilatory threshold (VT) by treadmill protocol (mean ± SD when available).

Author year	Peak V̇O_2_ (mL min^−1^ kg^−1^)	VT (mL min^−1^ kg^−1^) % peak V̇O_2_	*N*	Protocol	Time to exhaustion (min ± s)	Workloads at start, 5:00, and 10:00 relative to current study		
Current atudy	54.1 ± 4.6	37.8 ± 5.8 70.1 ± 10.7%	60	See Figure 1 5:00: 9.6 km h (6 mph) and 4% 10:00: 12 km h (7.5 mph) and 8%	10:33 ± 8			
Parpa and Michaelides (2022)	49.5 ± 5.6	Not reported	27	Start 4.8 km h (3mph) and 3% ↑ 1.2 km h min (0.7 mph) every 1:00 maintaining 3% At 3:00 ↑ 1.2 km h min (0.7 mph) every 2:00 maintaining 3% 5:00: and 3% 10:00: and 3%	13:30 ± 2:06		Speed	Grade
						Start	Lower	Lower
						5:00	Lower	Lower
						10:00	Lower	Lower
Lazic et al. (2019)	53.5 ± 4.8	47.5 ± 6.3	100	Reported as individual ramp	Not reported	Individual subject protocols		
Ponce‐González et al. (2015)	57.7 ± 5.5	51.9 90.4%	12	Warm‐up: 6 km h (3.5 mph) and 1% for 3:00 At 3:00 start 8 km h (5 mph) and 1% with ↑ 0.5 km h 30 s 5:00: 10 km h (6.2 mph) and 1% 10:00: 15 km h (9.3 mph) and 1%	Not reported	Start	Lower	Higher
						5:00	Higher	Lower
						10:00	Higher	Lower
Crisp et al. (2013)	50.0 ± 3.1	35.9 ± 4.1 71.8%	26	Start 8 km h (5 mph) ↑ 1 km h min 5:00: 12 km h (7.5 mph) 10:00: 17 km h (10.5 mph)	5:18 ± 1:39		Start	Lower
						5:00	Higher	Lower
						10:00	Higher	Lower
Boone and Bourgois (2013)	53.4 ± 4.8	Not reported	144	Start 8 km h (5 mph) Constant 1.5% ↑2 km h every 3‐min 5:00: 10 km h (6.2 mph) and 1.5% Once blood lactate = 4 mmol L; ↑1 km h and ↑ 0.5% every 30 s 10:00: unable to determine	Not reported	Start	Lower	Higher
						5:00	Higher	Lower
						10:00	Unknown	Unknown
Narazaki et al. (2009)	57.5 ± 8.2	Not reported	4	Start 4.8 km h (3 mph) ↑ 0.8 km h min 5:00: 8 km h (5 mph) 10:00: 12 km h (7.5 mph)	Not reported	Start	Lower	‐
						5:00	Lower	Lower
						10:00	Lower	Lower
Metaxas et al. (2009)	~ 49.6 ± 5.9	Not reported	61	Start 10 km h (6.2 mph) and 1% ↑ 2 km h 2 min 5:00: 12 km h (7.5 mph) and 1% 10:00: 18 km h (17.4 mph) and 1%	9:49 ± 00:46	Start	Higher	Higher
						5:00	Higher	Lower
						10:00	Higher	Lower
Sallet et al. (2005)	54.9 ± 7.2	No reported	58	5:00 warm‐up at 8.0 km h (5 mph) ↑ 1.5 km h (1 mph) every 2:30 until exhaustion 5:00: 8.0 km h (5 mph) 10:00: 11 km h (6.8 mph)	Not reported	Start	Lower	‐
						5:00	Lower	Lower
						10:00	Lower	Lower
Apostolidis et al. (2004)	51.7 ± 4.8	40.1; SD not reported 77.6 ± 7.0	13	Constant speed 10–12 km h (6.2–7.5 mph) ↑1.5% every 1:00 5:00: 10–12 km h (6.2–7.5 mph) and 6% 10:00: 10–12 km h (6.2–7.5 mph) and 13.5%	Not reported	Start	Higher	‐
						5:00	Unknown	Higher
						10:00	Lower	Higher
Caterisano et al. (1997)	53.4 ± 4.7	Not reported	17	4:00 self‐pace warm‐up Start 2 mph below self‐pace mile pace at 0% Speed increased to mile pace Then, 2% increase min 5:00: unable to determine 10:00: unable to determine	Not reported	Start	Unknown	Unknown
						5:00	Unknown	Unknown
						10:00	Unknown	Unknown
McInnes et al. (1995)	60.7 ± 8.6	Not reported	8	3:00 at 10 km h (6.2 mph), 12 km h (7.5 mph), and 14 km h (8.7 mph) At 9:00, ↑ 2% min 5:00: 12 km h (7.5 mph) 10:00: 14 km h (8.7 mph) and 2%	Not reported	Start	Higher	‐
						5:00	Higher	Lower
						10:00	Higher	Lower
Bolonchuk et al. (1991)	53.8 ± 4.5	Not reported	8	Bruce Protocol 5:00: 4.0 km h (2.5 mph) and 12% 10:00: 6.8 km h (4.2 mph) and 16% 20:00 8.8 km h (5.5 mph) and 20%	20:48 ± 1:24	Start	Lower	Higher
						5:00	Lower	Higher
						10:00	Lower	Higher
Parr et al. (1978)	46.0 ± 4.7	Not reported	34	Two protocols Used #1: 5.6 km h (3.5 mph) Start–3:00 8.8 km h (5.5 mph) 3:00–6:00 12 km h (7.5 mph) 6:00‐exhaustion Grade ↑ 2.5%/min except when speed changed 5:00: 8.8 km h (5.5 mph) and 7.5% 10:00: 12 km h (7.5 mph) and 17.5% #2: 6.4 km h (4.0 mph) Start–2:00 12 km h (7.5 mph) 2:00–4:00 12 km h (7.5 mph) 6:00‐exhaustion Grade ↑ 2.5%/2 min except when speed changed 5:00: 12 km h (7.5 mph) and 2.5% 10:00: 12 km h (7.5 mph) and 7.5%	Authors state 9:00–15:00	#1		
						Start	Lower	‐
						5:00	Lower	Higher
						10:00	Lower	Lower
						#2		
						Start	Lower	‐
						5:00	Higher	Lower
						10:00	Lower	Lower

The current study's V̇O_2_ at VT and VT percent of peak V̇O_2_ was 37.8 and 70.1.7%. In comparison, the four other VT investigations ranged from 35.9 to 51.9 for V̇O_2_ at VT and 71.8% to 90.4% for percent of peak V̇O_2_. These values, in a similar population, demonstrate great differences in VT and peak V̇O_2_ among studies. In addition to player fitness levels, it is possible the protocol may have affected the results.

### 4.4 Practical application

Cardiopulmonary exercise testing can be used to screen for unusually low CRF in an athlete due to an unknown pathology, deconditioning, lack of maintenance during the offseason, or assessing readiness to compete when coming back from injury. If a low CRF is discovered, an individual aerobic training program for the athlete can be designed.

The current study did not investigate the association of basketball specific metrics to VT or peak V̇O_2_. However, studies have measured in‐game HR and oxygen consumption, and advances in technology now allow collection of motion analysis (Abdelkrim et al., 2007; Narazaki et al., 2009). In‐game oxygen consumption is approximately 37^−1^ mL min kg^−1^ (65% of peak V̇O_2_) (Narazaki et al., 2009). Peak V̇O_2_ is correlated with mean V̇O_2_ during actual game time, as well as movements comprised of runs, jumps, and with repeat sprint ability (Stone & Kilding, 2009). This demonstrates practical application, as data gained from a CPET test may indicate how long and at what intensity level a player can play basketball.

Although a high peak V̇O_2_ is important, it is likely that the VT may be a better predictor of overall basketball performance (Krustrup et al., 2005; Sirotic & Coutts, 2007). Heart rates during a game average 169 bpm (Narazaki et al., 2009). The oxygen consumption and HR at VT in this study were 37^−1^ mL min kg^−1^ (70% of peak V̇O_2_) and 155 bpm. These values are almost precisely those previously reported as the in‐game values; a HR at VT of 155 bpm is between active in‐game and non‐active in‐game values. This implies that players in this study likely would be playing their active in‐game minutes at VT. This does not appear to be problematic, as exercise can continue above VT for short to moderate time periods, and basketball is an intermittent sport providing some periods of recovery every 2–3 min.

### 4.5 Limitations and future research

The DBTP was not tested against other protocols used in basketball players or that have been validated in non‐basketball subjects. Therefore, it cannot be stated that the DBTP is superior to other treadmill protocols for eliciting a true peak V̇O_2_. Different protocols, on the same players, need to be evaluated for eliciting true peak V̇O_2_ and VT. This study included men only, and future research should include women. Lastly, because no live game data were collected on V̇O_2_, HR, or motion analysis, we cannot relate the test‐associated measures to in‐game basketball performance.

## 5 CONCLUSION

The DBTP, and its predictive equation, is a valid protocol for use in the measurement of oxygen consumption in elite‐level basketball players. This may be a valuable tool when metabolic cart testing is not available. The measures of peak V̇O_2_, VT, and HRs from a CPET can be used to assess player fitness, design individual training protocols, and potentially help explain in‐game performance. Baseline values can be used as a reference standard when recovering from injury or illness. Further research that is integrated with new technology is needed toward this application to fully realize the potential.

## AUTHOR CONTRIBUTIONS

Brian D. Duscha: Designed experiment, tested subjects, performed analyses, prepared figures, drafted manuscript. William C. Bennett: Tested subjects, participated in editing manuscript. Jose Fonseca: Responsible for recruitment, scheduling, consenting, interpretation of data. Nicholas Potter: Responsible for recruitment, scheduling, consenting, interpretation of data. Kelsey N. Belski: Tested subjects, participated in editing manuscript. Megan A. Reaves: Tested subjects, participated in editing manuscript, metabolic cart, data interpretation. Brian J. Coyne: Participated in review of literature, manuscript editing/formatting. Annunziato Amendola: Coordination of study, interpretation of results. Aaron L. Baggish: Editing of manuscript, interpretation of results. Aaron L. Baggish: Principal Investigator, medical coverage for testing, interpretation of results, editing of manuscript.

## FUNDING INFORMATION

No external funding. Support was provided by Duke Sports Medicine Clinical Program Development.

## CONFLICT OF INTEREST STATEMENT

The authors declare that they have no conflicts of interest regarding the publication of this article. There are no financial conflicts of interest to disclose. The results of the study are presented clearly, honestly, and without fabrication, falsification, or inappropriate data manipulation.

## Data Availability

Data will be made available upon reasonable request.
